# Quinolines: the role of substitution site in antileishmanial activity

**DOI:** 10.3389/fchem.2025.1645334

**Published:** 2025-09-15

**Authors:** Orlando G. Elso, Guadalupe García Liñares

**Affiliations:** Laboratorio de Biocatálisis. Departamento de Química Orgánica y UMYMFOR, Facultad de Ciencias Exactas y Naturales, Universidad de Buenos Aires, Ciudad Universitaria, Buenos Aires, Argentina

**Keywords:** quinolines, antiprotozoal activity, substitution, antileishmanial agents, leishmaniasis

## Abstract

Leishmaniasis is one of the most widespread parasitic diseases in the world, primarily affecting the poorest and most vulnerable populations. The development of new therapeutic agents that are more efficient, safe, and selective remains a challenge. The quinoline framework emerges as a privileged scaffold for this purpose. This mini-review comprehensively analyses advancements from the last two decades on 2-, 3-, 6-, and 8-substituted quinolines, as well as polysubstituted analogues, as potential antileishmanial agents, focusing on how the position and nature of substituents influence their activity. Although the assays were conducted in different *Leishmania* species, 2- and 6-substituted quinolones generally show greater activity, often enhanced by the presence of halogen or hydroxyl groups.

## 1 Introduction

Leishmaniasis is the second most widespread protozoan disease globally. It is a parasitic infection caused by protozoa of the *Leishmania* genus; more than 20 species and subspecies can infect humans, leading to various clinical manifestations, from localized cutaneous leishmaniasis to visceral leishmaniasis, the more severe form, depending on the parasite species and the host’s immune system (García Liñares et al., 2006; Kumari et al., 2021). This disease is transmitted through the bite of infected female sandflies, and it is estimated that between 700,000 and one million new cases and between 26,000 and 65,000 deaths occur annually (World Health Organization, 2025).

The main drugs used to treat leishmaniasis are pentavalent antimonials, amphotericin B, and pentamidine (Pradhan et al., 2022; Silva Santos et al., 2020), but they present toxicity, primarily affecting the kidneys and heart (Brindha et al., 2021). Furthermore, pentamidine requires hospitalization for administration, which often leads to treatment discontinuation. Considering that: i) the complete eradication of the vector insects for leishmaniasis is infeasible; ii) the effective vaccines are not yet available; iii) the current chemotherapy is still deficient; and iv) there is a high level of resistance to these drugs, the search for new, more potent, selective, and safer compounds is essential.

In line with this, *N*-based heterocycles have received special attention due to a wide range of biological properties and pharmacological applications (Aatif et al., 2022; Heravi and Zadsirjan, 2020; Marshall et al., 2024). Since the discovery of the natural alkaloid quinine as an antimalarial drug, there has been great interest in the search for substituted quinolines as potential pharmacological agents (Yaluff et al., 2025). The relevance of quinolines is demonstrated in several reports regarding their use as antimalarial drugs (Ajani et al., 2022). Very recently, we have reported a revision on the use of quinoline antimalarial drugs as antileishmanial agents (Avanzo et al., 2025).

Particularly, quinoline-based compounds have arisen as a privileged scaffold for the development of more selective and potent antileishmanial agents. Based on currently used antimalarial drugs, the most significant development to date concerns 4-substituted quinolines (Delgado et al., 2025; Romero and Delgado, 2025). In addition to 4-substituted quinolines such as chloroquine, hydroxychloroquine, mefloquine, or amodiaquine, among others—well-known antimalarial drugs— (Avanzo et al., 2025; Ravindar et al., 2023), new compounds with various activities, such as antifungal (Vandekerckhove et al., 2013), antibacterial (Teng et al., 2018), or antitumor (Afzal et al., 2015), have also been developed. However, it has also been observed that quinolines substituted at other positions have also demonstrated antiparasitic activity (Nefertiti et al., 2018; Rajesh, 2018). Herein, we describe advancements from the last decades on the development of 2-, 3-, 6 and 8-substituted quinolines that display antileishmanial activity. Our focus is on these substitutions because they have yielded compounds with the most promising activity. Substitutions at other positions have been less frequently explored in the context of antileishmanial drug design and thus fall outside the scope of this analysis.

## 2 Substituted quinolines

### 2.1 2-Substitution

Given the antileishmanial activity of natural 2-substituted quinoline alkaloids (Fournet et al., 1993; 1996), a library of 2-substituted quinolines was synthesized (Fakhfakh et al., 2003; Franck et al., 2004). Initial *in vitro* screening against *L. amazonensis* and *L. infantum* identified quinolines substituted in the 2-position by a three-carbon alkenyl side chain containing an aldehyde (1), hydroxy (2) or bromine substituent (3) as the most active compounds, with IC_50_ values between 2 and 4 μM. By contrast, three carbon alkenyl side chains containing carboxylic acid, ester, amide, nitro or phenyl functionalities or larger alkenyl side chains displayed lower activity. To determine if this potent *in vitro* activity translated to a therapeutic effect, some derivatives were selected for *in vivo* studies. Compounds were administered by the oral route to a group of mice infected with *L. amazonensis*, *L. infantum*, and *L. donovani*. Interestingly, a disconnect was observed: compound 3, which was the most potent *in vitro*, showed no activity *in vivo*, whereas other derivatives with lower *in vitro* potency demonstrated a significant reduction (>80%) in parasite burden. Compounds 1 and 2 showed satisfactory activity in the visceral leishmaniasis models, with more than an 80% reduction of the parasite burden in the liver and close to 50% reduction in the spleen of mice infected with *L. infantum* and close to 50% reduction of the parasite burden in the liver of mice infected with *L. donovani* (Nakayama et al., 2005). Based on these results, the same group synthesized an α,β-unsaturated nitrile (4) with promising *in vitro* activity against *L. donovani* amastigotes and significant *in vivo* efficacy with a significant reduction in parasite burden in the liver (Nakayama et al., 2007).

The synthesis of a group of quinoline-2-carbohydrazides yielded compounds 5a and 5b as the most active against *Leishmania (Viannia) panamensis* (Coa et al., 2015). These compounds bear a 2-hydroxyphenyl moiety with a second hydroxy group at the 3- or 4- position of the aromatic ring, the presence of which is associated with the improvement of antileishmanial activity. Similar derivatives, with hydroxy groups replaced by methoxy functionalities, showed lower activity (Alodeani et al., 2015).

GDP-mannose pyrophosphorylase, which is involved in the biosynthetic pathway of glycoconjugates, has been recognized as an interesting target for the development of chemotherapeutic agents. Among several selected inhibitors, compound 6 emerged as the most promising antileishmanial agent, with an IC_50_ of 1.06 μM on axenic amastigotes and 0.63 μM on the RAW264.7 model of *L. donovani*. However, its selectivity index (SI) of 2.4 was low, limiting its therapeutic potential (Mao et al., 2017).

The primary strength of C-2 substitution lies in the accessibility for introducing different groups, which allows for an easy structural optimization. Despite this, a disconnection between *in vitro* potency and *in vivo* efficacy is generally observed.

### 2.2 3-Substitution

Based on previous studies describing the antileishmanial activity of 3-arylquinolines, this structure was selected as the lead to perform different substitution modifications at various positions (Katsuno et al., 2015; Ortiz et al., 2017). Some 3-substituted quinolines bearing alkenyl, alkynyl, and phenyl groups were synthesized and evaluated against *L. amazonensis* amastigotes. These analogues exerted low to negligible activity, suggesting this position is less favorable (Fakhfakh et al., 2003). Among a series of synthesized 3-arylamino quinolines, 3- and 4-fluorophenyl derivatives (7a-b) were effective against *L. mexicana* promastigotes (IC_50_ = 41.9 μM) with low cytotoxicity (>100 μM) (Chanquia et al., 2019).

Synthesis of hybrid molecules constitutes an attractive strategy for obtaining pharmaceutical compounds, which involves the covalent combination of two biologically active pharmacophores (Viegas-Junior et al., 2007). Several quinoline-based hybrids have been shown to exhibit various activities, including antimalarial, antibacterial, antiviral, antitumoral, and anti-inflammatory (Ammar et al., 2021; Hu et al., 2017; Singh et al., 2022). From this perspective, a library of quinoline-thiazolidinone hybrids was synthesized to target methionine aminopeptidase, an important enzyme for the development of antiprotozoal agents. Compound 8 arose as a promising antileishmanial agent with a twenty-fold higher inhibitory activity against *L. donovani* aminopeptidase (IC_50_ = 3.0 μM) compared to the human enzyme (IC_50_ = 58.0 μM), a good drug-likeness profile, and low cytotoxicity (CC_50_ > 150 μM) (Bhat et al., 2021).

Simple substitutions at C-3 have generally failed to produce potent compounds but the design of hybrid molecules with complex pharmacophores could yield more selective compounds. Additionally, a general lack of *in vivo* validation is observed.

### 2.3 6-Substitution

Substitution on the benzene ring also presents alternatives for the development of new drugs. Based on the concept of molecular hybridization, two series of 6-substituted quinolines linked to either oxadiazole-thiosemicarbazides or 1,3,4-thiadiazoles were synthesized. The *in vitro* activity against *L. major* promastigotes was evaluated, showing excellent antileishmanial activity with an IC_50_ in the submicromolar range. In the first series, fluorinated derivatives 9a and 9b were the most potent with IC_50_ values of 0.10 μM and 0.15 μM, respectively (Taha et al., 2017). In the second series, dihydroxyphenyl derivatives (10a-d) were the most potent compounds, achieving IC_50_ values as low as 0.04 µM (Almandil et al., 2019). Docking studies suggested a possible mechanism of action as these compounds are tightly fitted into the active site of pteridine reductase 1, a validated drug target in trypanosomatids. The presence and position of hydroxyl groups influenced antileishmanial activity, indicating that these groups afford polar interactions with residues from the active site of pteridine reductase 1. In the case of fluorinated derivatives, the one with the fluorine atom in the ortho position was the one that exhibited the most significant inhibition, demonstrating that the substitution in this position is essential for its activity.

The primary strength of the 6-substitution is the extremely high *in vitro* potency achieved through hybridization. In addition, the SAR is relatively clear, with electron-withdrawing and hydrogen-bond-donating groups significantly improving activity. The major limitation is the absence of amastigotes and cytotoxicity assays.

### 2.4 8-Substitution

The 8-position of the quinoline ring is historically significant due to the antimalarial drug primaquine. A group of *N*-quinolin-8-yl-arylsulphonamides showed promising activity (Da Silva et al., 2007). Particularly, the dihalogenated compounds 11a-c were very effective against both promastigotes of *L. amazonensis* and *L. chagasi*, and significant selectivity indices (SI). More lipophilic derivatives without halogenated substituents have shown somewhat lower activity. Promising results were obtained from assays on the amastigote form of *L. amazonensis*: significant antileishmanial activity was observed with compounds 11a-c, with IC_50_ < 1 µM. In contrast, substitution with a hydroxyl or phenyl group resulted in weaker antileishmanial activity against *L. amazonensis* or *L. (V) panamensis* amastigotes (Coa et al., 2020; Silva et al., 2018).

Chalcone, furochalcone, and chromone–quinoline hybrids via an alkyl linker have also been tested against *L. (Viannia) panamensis* (Coa et al., 2017; García et al., 2018). The antileishmanial activity was influenced by the length of the alkyl linker connecting the quinoline ring to its substituent. However, no clear correlation between antiprotozoal activity and alkyl chain length was established, as no consistent trend in activity relative to chain length was observed. Within the furanochalcone series, the derivative which features a three-carbon linker exhibited the highest activity against *L*. *(V) panamensis* amastigotes, but it showed high cytotoxicity. In the case of chromone-based series, compounds 12a and 12b, bearing two- and seven-carbon linkers respectively, showed the highest antileishmanial activity (IC_50_ = 16.9 and 17.0 μM). However, these compounds also displayed considerable cytotoxicity, with SI values below 1. By contrast, compounds 12c and 12d were less active but exhibited low cytotoxicity, with SI values of 4.89 and 5.84, respectively. All chalcone-quinoline hybrids showed moderate activity and low SI.

The low cytotoxicity constitutes the main strength of the 8-substitution pattern. Additionally, some compounds showed very potent activity against the clinically relevant amastigote form.

### 2.5 Polisubstitution

Considering the groups and substitution positions that have led to compounds with higher activity, new quinolines have been developed, combining different substituents at various positions. For example, a series of 2-styrylquinolines with additional groups at the C-7 position was synthesized and evaluated *in vitro* against the amastigote form of *L. donovani* (Loiseau et al., 2011). Compound 13 emerged as the most promising candidate, with IC_50_ = 1.2 µM and low toxicity (SI = 121.5). Additionally, it was observed that highly hydrophilic groups, such as COOH, dramatically decreased antileishmanial activity, whereas the presence of NO_2_ groups improved the SI. Compound 2 was taken as the lead structure for the synthesis of a series of quinolines with a hydroxypropenyl group at the two-position. *In vitro* and *in vivo* activity against *L*. *donovani* was measured (Gopinath et al., 2013). It was observed that the presence of halogens improved metabolic stability compared to the reference compound, and with a morpholine group at position 4, compound 14 emerged as the best candidate with significantly enhanced antileishmanial activity (IC_50_ = 0.22 μM, SI = 187). Furthermore, *in vivo* assays using a *L. donovani*/hamster model, its hydrochloride salt exhibited an 84% inhibition of parasite growth.

Considering the hybridization approach and the known antiparasitic activity of metronidazoles, a series of quinoline-metronidazole hybrids was synthesized and evaluated against *L. donovani* promastigotes and amastigotes (Upadhyay et al., 2019). Among these hybrids, compound 15 showed more potent activity against *L. donovani* both *in vitro* and *in vivo* and presented a high selectivity index and metabolic stability. Furthermore, the induction of apoptosis via mitochondrial membrane depolarization and an increase in the generation of reactive oxygen species was established as a possible dual mechanism of action. Compound 15 is a promising candidate both as a lead structure for the development of new antileishmanial agents and for clinical trials, taking into account its potency *in vitro* (IC_50_ = 3.75 µM in amastigotes) and *in vivo* assays (>80% reduction of parasite burden in liver and spleen), low toxicity (SI = 57.9) and excellent pharmacokinetic properties.

Consequently, a series of 3-aryl and 3-heteroaryl-*N* ^7^,*N* ^7^-dimethylquinoline-2,7-diamine derivatives was synthesized. These compounds were evaluated against *L. mexicana* amastigotes, showing promising activity (IC_50_ < 1 μM) (Hammill et al., 2021). According to SAR analysis, no significant variation in antileishmanial activity was observed upon substitution of the aromatic ring, and replacing the aryl group with six membered or bicyclic heterocycles also maintained the activity. However, substitution with five-membered heterocycles reduced activity, suggesting a minimum steric bulk requirement. Among the series, compounds 16 and 17 were the most active and were selected for *in vivo* experiments, but 16 exhibited toxicity, and 17 failed to suppress lesion progression in a murine model of cutaneous leishmaniasis.

Considering the antileishmanial activity of quinoline and 1,2,3-triazole scaffolds, a series of triazolyl 2-methyl-4-phenylquinoline-3-carboxylate derivatives was synthesized via click chemistry-based molecular hybridization approach and evaluated against *L. donovani* (Upadhyay et al., 2018). Despite good antileishmanial activity against promastigotes in most cases, only a few compounds showed significant inhibitory activity against intracellular amastigotes. Among them, compounds 18a-b and 19a-b were selected for their evaluation in a *L. donovani*/golden hamster model. Compounds 18a and 19a showed moderate activity, with 40% and 46% parasite inhibition, respectively, whereas 18b and 19b exhibited poor inhibitory activity.

Clioquinol (20), a dihalogenated 8-hydroxyquinoline, showed promising *in vitro* activity against *L. infantum* and *L. amazonensis* promastigotes and amastigotes, exhibiting low cytotoxicity against murine macrophages (CC_50_ = 834.4 µM) and human erythrocytes (CC_50_ = 1.5 × 10^3^ μM), with a higher selectivity index than amphotericin B. As no toxicity was observed when clioquinol was administered to BALB/c mice, it constitutes a promising candidate for subsequent *in vivo* studies (Tavares et al., 2018). Compound 21, a racemic 8-aminoquinoline derivative, has a strong effect on the mobility and morphology of *L. mexicana* promastigotes, exhibiting an IC_50_ value of 1.03 µM, markedly lower compared to glucantime (Isaac-Márquez et al., 2010). Also, 21 exhibited negligible cytotoxicity toward HeLa cells over 120 h.

The obvious strength of polysubstitution is the possibility of designing more active and selective molecules, optimizing pharmacokinetic and pharmacodynamic properties, although at the cost of increased synthetic complexity.

**FIGURE 1:**
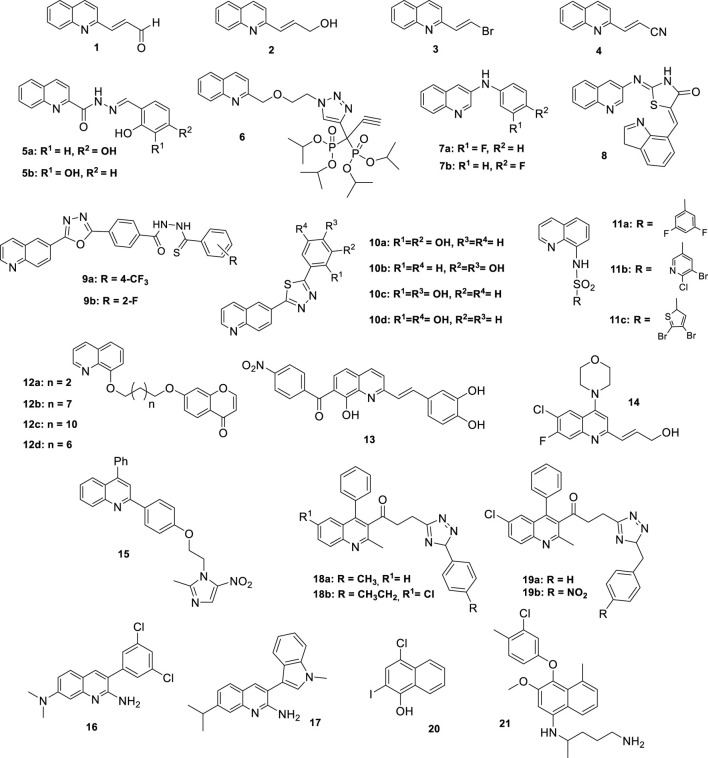
Structures of the more representative substituted quinolines with antileishmanial activity.

The structures of the most representative compounds are shown in the Figure 1 at the end of the manuscript. The Table 1 summarizes the antileishmania activity data of the most promising quinolines, which exhibit low IC_50_ values in *vitro* assays (<10 μM), or significant *in vivo* inhibition, or high selectivity indexes (SI > 10).

**TABLE 1:** Antileishmanial data for selected substituted quinolines.

Compound	*In vitro* evaluation	*In vivo* evaluation	Cytotoxicity	Selectivity index (SI)	Reference
*2-substitution*					
1	*L. amazonensis* IC_50_ = 4.0 µM (A) *L. infantum* IC_50_ = 2.0 µM (A)	No activity 93% reduction of parasite burden in the liver	CC_50_ = 9.0 µM	2.0 4.5	Fakhfakh et al. (2003) Nakayama et al. (2005)
2	*L. amazonensis* IC_50_ = 4.0 µM (A) *L. infantum* IC_50_ = 2.0 µM (A)	No activity 83% reduction of parasite burden in liver	CC_50_ = 34.0 µM	8.5 17.0	Fakhfakh et al. (2003) Nakayama et al. (2005)
3	*L. amazonensis* IC_50_ = 3.0 µM (A) *L. infantum* IC_50_ = 2.0 µM (A)	No activity	CC_50_ = 13.0 µM	4.3 6.5	Fakhfakh et al. (2003) Nakayama et al. (2005)
4	*L. donovani* IC_50_ = 2.4 µM (A)	83% reduction of parasite burden in liver			Nakayama et al. (2007)
5a	*L. (V) panamensis* IC_50_ = 2.6 µM (A)	ND	CC_50_ = 11.7 µM	4.5	Coa et al. (2015)
5b	*L. (V) panamensis* IC_50_ = 21.2 µM (A)	ND	CC_50_ = 99.6 µM	4.71	Coa et al. (2015)
6	*L. donovani* IC_50_ = 1.06 µM (A)	ND	CC_50_ > 100 µM	>94.3 (in BMDM)	Mao et al. (2017)
*3-substitution*					
7a	*L. mexicana* IC_50_ = 41.9 µM (A)	ND	CC_50_ > 100 µM	>2.4	Chanquia et al. (2019)
7a	*L. mexicana* IC_50_ = 41.9 µM (A)	ND	CC_50_ » 100 µM	» 2.4	Chanquia et al. (2019)
*6-substitution*					
9a	*L. major* IC_50_ = 0.10 µM (P)	ND	ND	-	Taha et al. (2017)
9b	*L. major* IC_50_ = 0.15 µM (P)	ND	ND	-	Taha et al. (2017)
10a	*L. major* IC_50_ = 0.04 µM (P)	ND	ND	-	Almandil et al. (2019)
10b	*L. major* IC_50_ = 0.08 µM (P)	ND	ND	-	Almandil et al. (2019)
10c	*L. major* IC_50_ = 0.7 µM (P)	ND	ND	-	Almandil et al. (2019)
10d	*L. major* IC_50_ = 0.9 µM (P)	ND	ND	-	Almandil et al. (2019)
*8-substitution*					
11a	*L. amazonensis* IC_50_ = 2.12 µM (P) *L. amazonensis* IC_50_ < 1 µM (A) *L. chagasi* IC_50_ = 0.45 µM (P)	ND	CC_50_ = 66.3 µM	31 >66 147	Da Silva et al. (2007)
11b	*L. amazonensis* IC_50_ = 2.25 µM (P) *L. amazonensis* IC_50_ < 1 µM (A) *L. chagasi* IC_50_ = 0.56 µM (P)	ND	CC_50_ = 67.4 µM	30 120	Da Silva et al. (2007)
11c	*L. amazonensis* IC_50_ = 2.85 µM (P) *L. amazonensis* IC_50_ < 1 µM (A) *L. chagasi* IC_50_ = 0.53 µM (P)	ND	CC_50_ = 68.7 µM	24 129	Da Silva et al. (2007)
12a	*L. (V) panamensis* IC_50_ = 16.9 µM (A)	ND	CC_50_ = 14.9 µM	0.89	Coa et al. (2017)
12b	*L. (V) panamensis* IC_50_ = 17 µM (A)	ND	CC_50_ = 9.5 µM	0.56	Coa et al. (2017)
12c	*L. (V) panamensis* IC_50_ = 34.2 µM (A)	ND	CC_50_ = 151.8 µM	4.9	Coa et al. (2017)
12d	*L. (V) panamensis* IC_50_ = 51 µM (A)	ND	CC_50_ = 290.8 µM	5.6	Coa et al. (2017)
Polisubstitution					
13	*L. donovani* IC_50_ = 0.22 µM (A)	84% inhibition of parasite growth (hydrochloride salt)	CC_50_ = 41.3 µM	187	Gopinath et al. (2013)
14	*L. donovani* IC_50_ = 1.2 µM (A)	ND	CC_50_ = 145.8 µM	122	Loiseau et al. (2011)
15	*L. donovani* IC_50_ = 5.42 µM (P) *L. donovani* IC_50_ = 3.75 µM (A)	>80% reduction of parasite burden in liver and spleen	CC_50_ = 217.2 µM	57.9	Upadhyay et al. (2019)
16	*L. mexicana* IC_50_ = 0.12 µM (A) *L. donovani* *BPK282* IC_50_ = 0.86 µM (A) *L. donovani* *BPK275* IC_50_ = 0.71 µM (A) *L. donovani* *BPK173* IC_50_ = 0.66 µM (A)	Showed toxicity	CC_50_ = 3.7 µM	31	Hammill et al. (2021)
17	*L. mexicana* IC_50_ = 0.22 µM (A)	No activity	CC_50_ = 3.7 µM	17	Hammill et al. (2021)
18a	*L. donovani* IC_50_ = 16 µM (P) *L. donovani* IC_50_ = 7 µM (A)	40.36%	CC_50_ = 39 µM	5.6	Upadhyay et al. (2018)
19a	*L. donovani* IC_50_ = 5 µM (P) *L. donovani* IC_50_ = 14 µM (A)	17.29%	CC_50_ = 99 µM	7.1	Upadhyay et al. (2018)
20	*L. infantum* IC_50_ = 4.7 µM (P) *L. infantum* IC_50_ = 3.2 µM (A) *L. amazonensis* IC_50_ = 8.3 µM (P) *L. amazonensis* IC_50_ = 6.2 µM (A)	ND	IC_50_ = 834.4 µM	177.1 260.1 99.9 135.6	Tavares et al. (2018)
21	*L. mexicana* IC_50_ = 1.03 µM (P)	ND	ND	-	Isaac-Marquez et al. (2010)

Note: promastigote (P), amastigote (A), ND, not determined.

In summary, in the last decades, the development of new quinoline-derived compounds as potential chemotherapeutic agents for the treatment of leishmaniasis has increased. This remains a significant challenge due to multiple causes, such as the numerous species that cause the disease, diverse manifestations, resistance, and more. The quinoline framework interacts with different receptors, ion channels, and enzymes, making it a privileged scaffold in organic chemistry for the search for bioactive compounds; many natural and synthetic derivatives have been shown to exhibit a wide range of biological properties. In this mini-review, we presented an overview of the impact of substitution position on the antileishmanial properties of substituted quinolines.

Considering all examples mentioned, it can be observed that, among monosubstituted quinolines, 2-substitution results in compounds with greater *in vitro* (amastigotes) and *in vivo* activity; 3-substituted quinolines are the least active compounds; and 8-substitution led to compounds with good activity *in vitro* on amastigotes and high SI, but dependent on *Leishmania* species. In many cases, for the same type of derivatives, the inclusion of hydroxyl or halogen groups leads to improved antileishmanial activity. In the case of polysubstituted quinolines, substituents in position 2 and/or a hydroxyl group in position 8 seem to be key in the antileishmanial activity and excellent selectivity index values. Among the compounds described, 2, 4, 6, 11a-c, 13, 14, 15, and 20 are the most promising either due to their low IC_50_ (<10 μM) values against amastigotes and/or high selectivity indexes (SI > 10) SI or good *in vivo* activity. However, a strict comparison is hindered due to several limitations, like: different *Leishmania* species with differences in drug sensitivity; in studies reporting very high potency on promastigotes, lack of data about assays against amastigotes, the clinically relevant form of the parasite; heterogeneity in experimental protocols; scarcity of information about molecular targets or action mechanisms. Future strategies should focus not only on a combination of substituents at diverse positions or synthesis of hydrid molecules, but also on systematic studies on structure-activity relationships and mechanisms of action.
